# 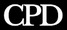 Improving hypertension control and cardiovascular health: An urgent call to action for nursing

**DOI:** 10.1111/wvn.12560

**Published:** 2022-02-08

**Authors:** Judith A. Hannan, Yvonne Commodore‐Mensah, Natsuko Tokieda, Alison P. Smith, Kate Sustersic Gawlik, Linda Murakami, Jennifer Cooper, Susan Koob, Kathy D. Wright, Doreen Cassarino, Cynthia Arslanian‐Engoren, Bernadette Mazurek Melnyk

**Affiliations:** ^1^ Division for Heart Disease and Stroke Prevention Centers for Disease Control and Prevention Chamblee Georgia USA; ^2^ Johns Hopkins School of Nursing Baltimore Maryland USA; ^3^ Division for Heart Disease and Stroke Prevention Centers for Disease Control and Prevention Chamblee Georgia USA; ^4^ Target: BP™ American Heart Association/American Medical Association Chicago Illinois USA; ^5^ College of Nursing The Ohio State University Columbus Ohio USA; ^6^ Practice Facilitation American Medical Association Chicago Illinois USA; ^7^ Association of Public Health Nurses Hood College Fredrick Maryland USA; ^8^ Preventive Cardiovascular Nurses Association Madison Wisconsin USA; ^9^ College of Nursing The Ohio State University Columbus Ohio USA; ^10^ American Association of Nurse Practitioners Austin Texas USA; ^11^ Million Hearts^®^ Sub‐Committee of the American Academy of Nursing Health Behavior Expert Panel School of Nursing University of Michigan Ann Arbor Michigan USA; ^12^ The Helene Fuld Health Trust National Institute for EBP Million Hearts^®^ Sub‐Committee of the American Academy of Nursing Health Behavior Expert Panel The National Forum for Heart Disease and Stroke Prevention The Ohio State University Columbus Ohio USA

**Keywords:** blood pressure, blood pressure measurement, cardiovascular disease, cardiovascular health, community interventions, health equity, healthcare disparities, hypertension, lifestyle coaching, nurses

## Abstract

**Background:**

Hypertension is a leading cause of cardiovascular disease (CVD) and affects nearly one in two adults in the United States when defined as a blood pressure of at least 130/80 mm Hg or on antihypertensive medication (Virani et al., 2021, *Circulation*, 143, e254). Long‐standing disparities in hypertension awareness, treatment, and control among racial and ethnic populations exist in the United States. High‐quality evidence exists for how to prevent and control hypertension and for the role nurses can play in this effort. In response to the 2020 *Surgeon General's Call to Action to Control Hypertension*, nursing leaders from 11 national organizations identified the critical roles and actions of nursing in improving hypertension control and cardiovascular health, focusing on evidence‐based nursing interventions and available resources.

**Aims:**

To develop a unified “Call to Action for Nurses” to improve control of hypertension and cardiovascular health and provide information and resources to execute this call.

**Methods:**

This paper outlines roles that registered nurses, advanced practice nurses, schools of nursing, professional nursing organizations, quality improvement nurses, and nursing researchers can play to control hypertension and prevent CVD in the United States. It describes evidence‐based interventions to improve cardiovascular health and outlines actions to bring hypertension and CVD to the forefront as a national priority for nursing.

**Linking Evidence to Action:**

Evidence‐based interventions exist for nurses to lead efforts to prevent and control hypertension, thus preventing much CVD. Nurses can take actions in their communities, their healthcare setting, and their organization to translate these interventions into real‐world practice settings.

## INTRODUCTION

Cardiovascular disease (CVD) remains the leading cause of morbidity and mortality for both men and women across the United States and worldwide (GBD 2017 Causes of Death Collaborators, 2018). Hypertension, a leading cause of CVD, affects nearly one in two adults in the United States when defined as having a blood pressure (BP) of at least 130/80 mm Hg or taking antihypertensive therapy (Virani et al., 2021). Yet, in a recent study, only 77% of individuals were aware that they had hypertension, and only 44% of those with hypertension had their BP controlled to <140/90 mm Hg in 2018 (Muntner et al., 2020). Uncontrolled hypertension is an independent risk factor for CVD, stroke, kidney disease, and cognitive decline and significantly contributes to complications of pregnancy and mortality globally (U.S. Department of Health and Human Services, 2020b; Whelton et al., 2018; Williamson et al., 2019).

Hypertension is mostly preventable and controllable (Mills et al., 2020; GBD 2017 Risk Factor Collaborators, 2018). In the United States, progress in improving hypertension control has stalled over the last decade. Without improvements in healthy lifestyle behaviors and healthcare delivery, the decade‐long improvements in cardiovascular health will erode (Kirkland et al., 2018). The ongoing COVID‐19 pandemic has disrupted preventive care and has presented barriers to hypertension control, including worsening mental health and unhealthy coping mechanisms such as unhealthy eating and decreased physical activity (Bhutani et al., 2021; Czeisler et al., 2020).

Long‐standing disparities in hypertension awareness, treatment, and control among racial and ethnic populations in the United States must be addressed (Centers for Disease Control and Prevention [CDC], 2021; Commodore‐Mensah et al., 2021; Lackland, 2014). Ample evidence exists for prevention and control of hypertension (Appel, 2003; Carey et al., 2018; Whelton et al., 2018), as well as for the role of nurses in preventing and managing hypertension (Himmelfarb et al., 2016; Proia et al., 2014).

In response to the trend of worsening hypertension control, the 2020 *Surgeon General's Call to Action to Control Hypertension* identified three broad goals: (1) Make hypertension a national priority, (2) Ensure places where we live, work, and play support hypertension control, and (3) Optimize patient care for hypertension control (U.S. Department of Health and Human Services, 2020a).

### Aim

Within 2 months of the publication of the *Surgeon General's Call to Action*, individuals from public health nursing, cardiovascular nursing, community health center nursing, nursing and medical associations, and academia formed a workgroup to develop a “Call to Action for Nurses” to improve control of hypertension and to make sure nurses can get the information and resources they need to execute this call to action.

Workgroup members reviewed the literature, synthesized the evidence, and made recommendations. This report delineates the critical role of nursing in improving hypertension control in the United States, highlights evidence for nursing interventions to improve hypertension control and cardiovascular health, and describes information and resources nurses can use to improve hypertension control.

## A VISUAL TOOL SHOWING SPECIFIC ACTIONS THAT NURSING GROUPS CAN TAKE

**FIGURE 1:**
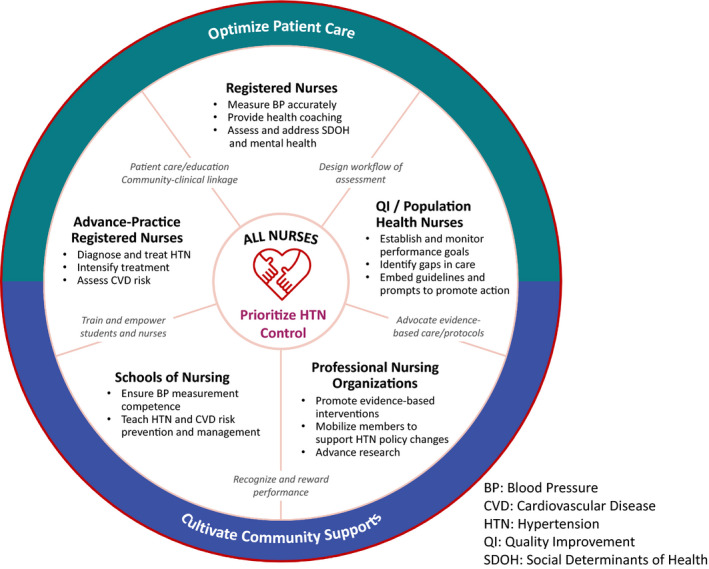
Nursing actions to improve hypertension control and CVD risk reduction. *Note*. BP, blood pressure; CVD, cardiovascular disease; HTN, hypertension; QI, quality improvement; SDOH, social determinants of health

Figure 1 represents a unifying call to action for all nurses and shows the specific actions that various nursing sectors can take, keeping the individual with hypertension at the center of their actions. Figure 1 provides nurses an easy way to see what each group can do. Following Figure 1 is detail on the potential actions nurses can take, broken down by nursing groups or sectors.

## WHAT THIS CALL TO ACTION MEANS FOR REGISTERED NURSES (RNs)

Numbering almost 4 million in the United States, RNs are the largest segment of the healthcare workforce (American Association of Colleges of Nursing, 2019) and serve in a wide range of settings: acute care, community health centers, public health agencies, primary care practices, home health care, nursing homes, and other settings.

Accurate BP measurement, a critical step in hypertension diagnosis and treatment decision, is a fundamental nursing skill. When RNs do not measure BP accurately, it can lead to over/under diagnosis, over/under‐treatment, or lack of treatment intensification (Bundy et al., 2017; Johnson et al., 2018; Jones et al., 2003; Piper et al., 2015). The most common cause of clinical inertia (i.e., failure of a clinician to intensify treatment) is the concern that a BP is not representative of a patient's true BP, either because it is inaccurately measured or because of an insufficient number of readings. Thus, RNs need to be meticulous about accurate BP measurement, whether using a manual or an automated BP cuff (Muntner et al., 2019).

Specific actions for RNs include the following:

- Be re‐skilled every 6–12 months as recommended by the 2019 American Heart Association (AHA) Scientific Statement on the Measurement of BP in Humans (Muntner et al., 2019).
- Ensure proper positioning of patients in a supportive chair (rather than on an exam table).
- Ensure that the environment allows an accurate measurement (Pickering et al., 2005).
- Educate certified nursing assistants, medical assistants, and others in accurate BP measurement and technique (Block et al., 2018).
- In practice settings, develop protocols for when and how BP is measured and address abnormal results during healthcare encounters (Josiah Macy Jr. Foundation, 2017).
- In the community, collaborate with businesses, pharmacies, and payers to ensure BP monitors and screening locations are available outside of the healthcare setting.
- Review data from electronic health records to identify and address untreated or uncontrolled BP.

Evidence supports self‐measured blood pressure (SMBP) monitoring (Shimbo et al., 2020), so RNs should educate and motivate patients to self‐monitor their BP. This includes the following:

- Teaching patients to accurately use and calibrate their home BP monitors (Parati et al., 2021).
- Using motivational interviewing in serving as a health coach (Hickey et al., 2019).

RNs can refer patients to evidence‐based programs for preventing or managing chronic disease such as Stanford's Chronic Disease Self‐Management Education (Administration for Community Living, 2021), the CDC’s National Diabetes Prevention Program (CDC, 2019), and others.

RNs can identify social and structural determinants of health that are barriers to seeking preventive screenings and health care.

For Black or African American adults, the experience of racial discrimination increases the incidence of hypertension by 50% (Forde et al., 2020). Black or African American adults are more likely to have uncontrolled hypertension than other racial and ethnic groups (Carnethon et al., 2017). Black or African American and Hispanic adults have a higher lifetime risk of hypertension than White adults (Carson et al., 2011; Fryar et al., 2017). Nearly 50% of Black or African American women with uncontrolled hypertension have depression (Gabriel et al., 2021).

RNs can address social determinants of health (SDOHs) that affect hypertension, including access to transportation, health care, medication, safe places to exercise, and nutritious food. They can:

- Establish open clinic hours so that patients who rely on public transportation will not be penalized for late clinic appointments.
- Screen for SDOH using the accountable health communities screening tool (Billioux et al., 2017; Casey et al., 2019); the Protocol for Responding to and Assessing Patients' Risks, Assets, and Experiences (Weir et al., 2020); or other standardized screening tools.
- Work with local partners such as community health workers, social service, and other community‐based support agencies that are poised to help address SDOH.
- Establish an inventory of the above local community resources that promote clinical and community linkages and advance health equity (Ibe et al., 2021).

Attending to the above actions, RNs will be influencing more than control of hypertension, as such initiatives can help eliminate health disparities.

## WHAT THIS CALL TO ACTION MEANS FOR ADVANCED‐PRACTICE REGISTERED NURSES (APRNs)

Nurse practitioners (NPs) are advanced practice RNs (APRNs) who blend clinical expertise in diagnosing and treating health conditions, including hypertension, with an emphasis on disease prevention and providing health education, health coaching, and counseling to their patients. As of December 2020, more than 325,000 NPs were licensed to practice in the United States, providing more than 1 billion patient visits each year (American Association of Nurse Practitioners, 2021). Specific actions for APRNs include the following:

- Routinely screening and diagnosing hypertension through accurate BP measurement.
- Providing patient education on the importance of hypertension prevention, control, and evidenced‐based ways to adopt and sustain a heart‐healthy lifestyle.
- Prescribing antihypertensive medications using established guidelines such as the 2017 ACC/AHA Hypertension Guideline (Whelton et al., 2018).
- Routinely screening for and providing early evidence‐based interventions for depression and anxiety/stress given the evidence showing the association between these factors and CVD (Gawlik et al., 2019; Giannoglou & Koskinas, 2015; Levine et al., 2021).
- Screening for other common comorbidities such as diabetes and initiating evidence‐based treatment regimens as needed.
- Using motivational interviewing to assist patients with healthy lifestyle behavior change (Sawyer et al., 2020).
- Ensuring adequate treatment, timely follow‐up, treatment intensification, and, if needed, referrals to specialists until BP goals are reached.
- Regularly assessing for side effects of hypertension medication, adherence to antihypertensive therapy, and lifestyle changes.
- Educating colleagues who measure BP in their clinics to ensure accurate BP measurement.

## WHAT THIS CALL TO ACTION MEANS FOR SCHOOLS OF NURSING

Schools of nursing must ensure their graduates can execute the aforementioned strategies designed to improve BP control. They should teach the importance of early and accurate diagnosis, evidence‐based healthy lifestyle interventions, behavior change strategies to prevent CVD, assessments and steps for addressing SDOH, and team‐based care. Specific actions for schools of nursing include the following:

- Teaching accurate BP measurement at multiple points across curricula.
- Ensuring competence of students in conducting cardiovascular and mental health assessments.
- Incorporating the latest evidence‐based guidelines on exercise, nutrition, wellness, stress, anxiety and depression management, and BP diagnosis and management.
- Teaching RNs and APRNs to incorporate SMBP monitoring and healthy lifestyle prescriptions into patient care and management.
- Preparing nurses to educate patients about their CV risks, diagnosis of hypertension, treatment regimen, and adherence strategies.
- Enable students and nurses to exercise, choose healthy foods, and get care for mental health concerns.
- Integrating the Million Hearts^®^ Fellowship module in nursing education and public health nursing and providing students with hands‐on experience in cardiovascular screenings and cardiovascular risk reduction counseling (Gawlik & Melnyk, 2015).
- Including team‐based care, evidence‐based quality improvement, and population health management techniques in nursing curricula.
- Implementing a standardized, evidence‐based treatment protocol that university health centers can use to identify and treat faculty, staff, and students with hypertension.
- Teaching about SDOHs and their role on cardiovascular outcomes, how to complete a SDOH screening, and how to find and connect patients to local community resources.
- Examining the impact of structural racism/discrimination on hypertension control and mitigation of unconscious bias in the healthcare setting.
- Incorporating the assessment of depression and anxiety into curricula because these can be barriers to hypertension control.
- Emphasizing population health cardiovascular prevention including community‐based interventions, such as establishing walkable communities, and access to healthy food options.
- Teaching students how to stay current on the best and latest evidence to prevent and manage hypertension and CVD through education and skills building in evidence‐based practice (Melnyk & Fineout‐Overholt, 2019).
- Ensuring that students meet the evidence‐based practice competencies for RNs and APRNs by the time they graduate from their academic programs (Melnyk et al., 2014, 2016).

## WHAT THIS CALL TO ACTION MEANS FOR PROFESSIONAL NURSING ORGANIZATIONS

Professional nursing organizations are crucial to improving hypertension control and promoting overall cardiovascular health. These organizations include the American Nurses Association, the American Academy of Nursing, the Preventive Cardiovascular Nurses Association, the National Black Nurses Association, the American Association of Nurse Practitioners, and CV nursing affinity groups like the AHA Council on Cardiovascular and Stroke Nursing, among others. Professional nursing associations’ members can help influence their healthcare organizations and communities by sharing messaging to improve hypertension prevention, diagnosis, and treatment through education and leadership. These organizations also should prioritize actions supporting nurses adopting healthier lifestyles and attending to their cardiovascular health. Specific actions for professional nursing organizations include the following:

- Making the topic of cardiovascular health a priority for national conferences and meetings.
- At meetings, allowing for frequent recovery breaks and movement and encouraging healthy meals and snacks.
- Encouraging nurses to make healthy lifestyle choices and to prioritize self‐care.
- Promoting evidence‐based interventions and strategies to improve hypertension diagnosis and management as well as cardiovascular risk reduction.
- Encouraging members to be leaders in advocating for community interventions that lead to better cardiovascular health, such as those found in the “Guide to Community Preventive Services” (Community Preventive Services Task Force, n.d.).
- Showcasing best practice protocols for hypertension control in newsletters, journals, and other publications.
- Developing programs that recognize and reward nursing actions to improve hypertension control.
- Creating and disseminating educational resources on the prevention, diagnosis, and treatment of hypertension to be used by healthcare professionals and patients.
- Educating nurses on SDOHs and barriers to care that affect people's ability to prevent, diagnose, treat, and manage their hypertension.
- Developing evidence‐based interventions to mitigate racism, discrimination, and structural racism in the healthcare setting, such as reviewing historical practices and policies to ensure all patients are treated equally.
- Supporting initiatives to educate members, patients, and the general public on hypertension control and lifestyle choices that promote health.
- Advancing research in hypertension prevention and treatment.
- Integrating hypertension diagnosis and management curricula into continuing education programming.
- Reinforcing BP measurement competency through training and certification programs every 6–12 months.
- Generating and supporting national campaigns to educate the public on hypertension management and prevention of CVD.
- Championing and encouraging insurers to consider covering services related to heart health. These include the following:
  - Coverage of validated SMBP devices.
  - Coverage of medication, including combination pills.
  - Reimbursement for patient education regarding diagnosis, medication use, SMBP monitoring training for technique, and reporting results back to clinician, including technology assistance if needed.
  - Coverage of lifestyle change programs.
  - BP rechecks without co‐pay.
  - Synchronization of and multi‐month medication refills without additional co‐pays.
  - Reimbursement for cardiovascular risk reduction education by nurses.

## WHAT THIS CALL TO ACTION MEANS FOR EVIDENCE‐BASED QUALITY IMPROVEMENT AND POPULATION HEALTH NURSES

With additional education and skills in evidence‐based quality improvement (EBQI) and population health, nurses can play a pivotal role in improving BP control within healthcare systems or populations using data to drive lasting change (Melnyk et al., 2015; Melnyk & Morrison‐Beedy, 2019). Nurses can do this using clinical practice facilitation within a specific ambulatory care setting, managerial EBQI roles within a health system, population management programs through a payer or EBQI collaborative, and public health initiatives at the local, state, and national level. Regardless of the level of involvement, nurses are skilled in team‐based care and EBQI principles requiring them to identify and engage leadership, clinical champions, and other colleagues—including primary care nurses connecting to specialty care nurses and nurses connecting to colleagues from medicine, pharmacy, social work, biomedical engineering, health information technology, and other stakeholders.

With the support from leadership making hypertension control an institutional priority and engaging a team of supportive stakeholders, health systems can equip EBQI nurses with evidence‐based tools and resources to drive practice change and improve patient outcomes (Casey et al., 2021). Examples of evidence‐based tools can be found in Tables S2–S5.

Specific actions that EBQI nurses can take include the following:

- Using practice data, such as a hypertension registry (Whelton et al., 2018), to quantify gaps in care and establish baseline performance for indicators such as follows:
  - Rates of hypertension control, including rates by subpopulation such as gender, race and ethnicity, or age, as well as by individual clinician or care location.
  - Frequency of follow‐up visits for patients with uncontrolled hypertension.
  - Rates of treatment intensification for patients with uncontrolled hypertension.
  - Rates of lifestyle interventions.
  - Rates of response to treatment for patients who have had a change in their treatment.
  - Rates of medication refills.
  - Reporting on the frequency with which patients are using SMBP measurement.
- Establishing and monitoring performance goals, including performance, process quality, and structural measures (Casey et al., 2019), such as follows:
  - Performance measures, including control rates at various stages of hypertension.
  - Process quality measures, including intervention, adherence, and use of SMBP
  - Structural measures including use of standardized protocols for measurement accuracy, Atherosclerotic Cardiovascular Disease risk assessment, shared decision‐making, and team‐based care.
- Elevating clinical practice guidelines and evidence to drive continuing professional education, policies, and protocols (Whelton et al., 2018).
- Embedding evidence‐based guidelines and prompt adherence to protocols like confirmatory measurements and treatment intensification for patients with uncontrolled BP in health information technology (Whelton et al., 2018). A nursing informaticist is well‐suited for this role.
- Facilitating team huddles to identify patients with uncontrolled BP and considering assessments and interventions to achieve BP goals.
- Educating care teams on evidence‐based care.
- Serving as clinical practice facilitators coaching care teams in ambulatory care settings to adopt and adhere to evidence‐based, systematic care practices.

Evidence of nurses playing a role in EBQI includes examples such as follows:

- Implementation of nurse‐led community‐based chronic disease management models to engage low‐income urban residents in BP management (Sanders & Guse, 2017).
- Dissemination of SMBP monitoring protocols to patients to assure accurate BP measurement in the home (Parati et al., 2021).

## CONTRIBUTIONS FROM AND CONSIDERATIONS FOR NURSE RESEARCHERS

Nurse researchers play an essential role in designing and conducting trials to improve hypertension outcomes. In 1976, the Taskforce on the Role of Nursing in High Blood Pressure Control affirmed the importance of conducting research to increase knowledge of nursing interventions targeted at improving hypertension control (National Institutes of Health, 1976). Nurses have since led trials that have provided the evidence base for clinical practice and trials focused on community‐based interventions to improve hypertension control.

Exemplars of nurse‐led clinical trials to improve hypertension control include the Community Outreach and Cardiovascular Health Trial (Allen et al., 2011), Nurse‐Managed BP Telemonitoring Among Urban African Americans (Artinian et al., 2007), and the Self‐Help Intervention Program for High Blood Pressure Care (Kim et al., 2011).

Several hypertension trials have included a nursing intervention as part of a multi‐component intervention, as described in Table S1.

Nurse researchers should address current challenges in cardiovascular health and hypertension control, prioritizing research where there is not enough evidence to guide practice. Nursing researchers should also collaborate with nursing faculty and Doctor of Nursing Practice prepared nurses to ensure research findings are implemented into clinical practice without delay. This research‐practice time gap is estimated to be 15 years (Khan et al., 2021). Priority topics for cardiovascular and hypertension research should include the following:

- Lifestyle interventions to prevent the development of hypertension in high‐risk populations.
- Interventions to reduce anxiety, stress, and depression in people with CVD.
- Examination of underlying mechanisms of health behavior change.
- Culturally informed interventions to promote healthy behaviors.
- Developing and testing interventions to improve hypertension control and cardiovascular risk, including healthy lifestyle behavior change.
- Health disparities and CVD.
- Maternal health interventions to promote cardiovascular health.
- Interventions that address SDOHs and improve hypertension control.
- SMBP monitoring to reduce hypertension disparities.
- Interventions to mitigate structural racism and discrimination to increase access to care.
- Ways that informatics nurses, statisticians, and computer scientists can work together to turn big data into information that can be translated into nursing practice.
- Dissemination and implementation of studies to speed the translation of evidence‐based interventions to control hypertension and prevent CVD into real‐world clinical settings.
- How structural racism contributes to poor hypertension control and cardiovascular health and interventions to mitigate the effects of structural racism.

## RESOURCES THAT NURSES FROM ALL SECTORS CAN USE

Preventing hypertension and improving hypertension control is well understood. Over the last decade, nurses have been the source of major national organizations and initiatives to improve prevention and control of hypertension. These organizations and initiatives provide tools and resources to improve control of hypertension; this paper presents these resources.

Million Hearts^®^ is a national initiative co‐led by the CDC and Centers for Medicare & Medicaid Services to prevent 1 million heart attacks, strokes, and other cardiovascular events over a 5‐year period. It focuses on implementing a small set of evidence‐based priorities and targets that can improve cardiovascular health for all. The Million Hearts^®^ website offers numerous tools for patients and providers and describes evidence‐based strategies to decrease physical inactivity, tobacco use, and salt intake. It outlines care changes needed to improve use of aspirin as appropriate, BP control, cholesterol management, and smoking cessation. It lists key actions needed to improve outcomes of specific priority populations. Nurses can join partner meetings and specific forums and read newsletters on the site.

Target: BP™ is a national initiative of the American Heart Association and the American Medical Association (AMA) in response to the high prevalence of uncontrolled BP. It helps healthcare organizations and care teams, at no cost, improve BP control rates through an evidence‐based quality improvement program and recognizes organizations committed to improving BP control.

The Preventive Cardiovascular Nurses Association (PCNA) is a nursing organization preventing cardiovascular disease through assessing risk, facilitating lifestyle changes, and guiding individuals to achieve treatment goals. PCNA is committed to the continued education and support of nurses so they may successfully rise to this challenge. PCNA offers a Cardiovascular Nursing Certificate program and numerous resources for patient education and for providers.

The AMA leads and participates in numerous efforts to improve control of hypertension. Their “About Improving Health Outcomes” web page has specific BP resources to assist healthcare practices and clinicians in building a team to focus on hypertension to measure accurately, act rapidly, partner with patients, and promote appropriate use of SMBP monitoring.

The websites of all four of these organizations offer numerous tools to assist nurses. Tables S2–S5 provide detailed lists and links to specific resources from these and other organizations sorted by the three goals of the *Surgeon General's Call to Action*: (1) Make hypertension control a national priority, (2) Cultivate community supports, and (3) Optimize patient care for hypertension. Given the need for all nurses to be able to provide an accurate assessment of BP, we included a fourth table with resources specific to accurate measurement.

## LINKING EVIDENCE TO ACTION

- Nurses in all settings should emphasize lifestyle modification, an evidence‐based strategy to prevent, treat, and control hypertension.
- Nurses should link patients to peer support programs for physical activity and nutrition or advocate for others to do so.
- Improving food purchasing practices and walkability of communities are important to general health and to preventing and managing hypertension.
- Assessing and addressing SDOHs and psychological health can reduce health disparities in vulnerable populations.
- Nurses should ensure accuracy of BP measurement, as this is a critical step in hypertension diagnosis, treatment, and control.
- Patients should be taught to measure their BP.
- Evidence‐based guideline‐recommended care, comprehensive treatment protocols, self‐measured blood pressure monitoring, and team‐based care are all important.

## CONCLUSIONS

Uncontrolled hypertension causes CVD, stroke, and kidney disease, among other health problems, which cause unnecessary human suffering and premature death. Disparities in hypertension diagnosis, treatment, and control among racial and ethnic groups in the United States should be addressed. We know what to do to prevent the development of hypertension, detect it early, and control it, yet, as a nation, we are not succeeding. Nurses are well positioned to contribute meaningfully to national efforts to improve hypertension control, using the ample evidence on how to prevent, diagnose, treat, and control hypertension.

The COVID‐19 pandemic has placed demands on nurses and the nursing profession, but as nurses, we know that our colleagues, faculty, and students can still be inspired by a new call to action to save lives. Our ask is to use the evidence and resources and seize opportunities to act. More information about how to join with other nurses in this call to action can be found at https://millionhearts.hhs.gov/.

## Supporting information

Table S1‐S5
