# Supplementary material for: [image] Improving hypertension control and cardiovascular health: An urgent call to action for nursing
Source: Worldviews Evid Based Nurs. 2022 Feb 8;19(1):6–15. doi: 10.1111/wvn.12560 (PMC9305122; doi:10.1111/wvn.12560)
Supplement: Supplementary file 1 — Table S1‐S5 [file WVN-19-6-s001.docx]

**Table S1.** Examples of Nurse-Led Interventions in Studies Addressing Hypertension Control

| Study title | Nursing intervention |
| --- | --- |
| Nurse-Led Interventions for Hypertension (Spies, Bader, Opollo, & Gray, 2018) | Nurses provide 80% of health care in East Africa, and their interventions are relevant to implementing or improving hypertension screening, diagnosis, and treatment. The nurse-led interventions also increase access to care and are cost-effective. |
| Hypertension Intervention Nurse Telemedicine Study (HINTS) (Bosworth et al., 2007) | Registered Nurses (RNs) (supervised by a primary care physician) implemented recommendations based on home blood pressure (BP) values. Nurses administered tailored behavioral intervention, medication management according to a hypertension decision support system, or a combination of both. |
| Community Outreach and Cardiovascular Health [COACH Trial] (Allen et al., 2011) | The nurse practitioner (NP) and community health worker worked as a team to deliver a behavioral intervention to improve therapeutic lifestyle changes and adherence to medications and appointments. The NP followed an algorithm for the treatment of hypertension, diabetes, and hyperlipidemia. |
| Nurse-Managed Blood Pressure Telemonitoring with African Americans (Artinian et al., 2007) | RNs delivered the home blood pressure monitor and trained the participants in self-measured blood pressure (SMBP) monitoring. The RN provided telecounseling on lifestyle modification and medication adherence. |
| Nurse-led task shifting strategy for hypertension control (TASSH) (Ogedegbe et al., 2018) | Community health nurses delivered the World Health Organization cardiovascular disease (CVD) package, which included clinical decision support for management of CVD via easy-to-follow algorithms, lifestyle counseling, and drug treatment protocols. The nurses initiated antihypertensive therapy for participants with low/medium CVD risk using a treatment algorithm and referred patients with CVD risk to district hospitals. |
| Phone-Based Intervention Under Nurse Guidance After Stroke (PINGS) Trial (Sarfo et al., 2019) | RNs trained stroke survivors in SMBP monitoring and followed up with a phone call if mean BP exceeded thresholds. The RN assessed medication adherence and used motivational interviewing to address barriers to medication adherence. |
| Mindfulness and the Dietary Approaches to Stop Hypertension (DASH) in Older Adults With Mild Cognitive Impairment (Wright et al., 2021) | The nurse led an interprofessional team to deliver mindfulness plus a culturally-tailored version of the DASH diet to improve systolic blood pressure and stress. Participants were divided into six groups randomized 1:1:1 to the mindfulness DASH group, attention only (non-hypertensive education) group, or true control group. The mindfulness DASH and attention only interventions were delivered in eight weekly 2-hour group sessions. The mindfulness in motion included movements from chair/standing, breathing exercises, and guided meditation. The DASH diet component used a critical thinking approach of problem solving, goal setting, reflection, and self-efficacy. |
| Home and Online Management and Evaluation of Blood  Pressure (HOME BP) (Band et al., 2016; McManus et al., 2021) | The nurse prescriber or general practitioner conducted a blood pressure drug review and selected an individualized drug titration plan. Practice nurses provided additional behavioral support for SMBP monitoring and lifestyle modification using a motivational approach (congratulate, ask, reassure, encourage). |
| Home blood pressure monitoring with nurse-led telephone support (Kerry et al., 2013) | The nurse provided SMBP monitoring support and telephone support until the blood pressure target was reached. |

*Note.* References are on the last page of this document.

**Table S2.** Resource List Related to “Prioritize Control Nationally”

| **Goal 1. Make hypertension control a national priority.** | | | |  |
| --- | --- | --- | --- | --- |
| ***Strategy A. Increase awareness of the health risks of uncontrolled hypertension.*** | | | |  |
| Resource (link) | Author/  Sponsor | Type of resource | Description | |
| Mind Your Risks Campaign  (<https://www.mindyourrisks.nih.gov/>) | NINDS | Campaign resources, including social media graphics, related links | Public health campaign educating people with, or at risk of, high blood pressure using social media and resources. | |
| Manage your BP  (<https://www.adcouncil.org/campaign/high-blood-pressure-control>)  (<https://www.heart.org/en/health-topics/high-blood-pressure/commit-to-a-plan-to-lower-your-blood-pressure>) | Ad Council, AHA, AMA, ASA | Campaign resources, including education tools | Public awareness campaign, covering free multimedia to raise awareness regarding risk of high blood pressure for heart attack and stroke. | |
| Hypertension Patient Education: Tools and Handouts  (<https://pcna.net/clinical-resources/patient-handouts/hypertension-patient-tools-and-handouts/>) | PCNA | Clinical education tool for patients (booklet), a fact sheet | Education for clinicians to use with patients with high blood pressure. | |
| Blood Pressure Patient Resources  (<https://pcna.net/clinical-resources/patient-resources/blood-pressure/>) | PCNA | Patient education webpage | Education for patients and caregivers about how to check and control blood pressure. | |
| Release the Pressure campaign (<https://releasethepressure.org/>) | Ad Council, AHA, AMA, AMA Foundation, ABC, NMA, MHI | Campaign resources, including education, tools, and recommendations | Public awareness campaign empowering African American women to take control of their blood pressure through education and motivation. | |
| ***Strategy B. Recognize the substantial economic costs of uncontrolled hypertension.*** | | | |  |
| Resource (link) | Author/  Sponsor | Type of resource | Description | |
| Costs & Consequences (<https://millionhearts.hhs.gov/learn-prevent/cost-consequences.html>) | Million Hearts® | Key facts | Statistical data about the associations between costs and heart disease and stroke. | |
| ***Strategy C. Eliminate disparities in the treatment and control of hypertension.*** | | | |  |
| Resource (link) | Author/  Sponsor | Type of resource | Description | |
| Ethnicity & Disease - Social Determinants of Health as Potential Influencers of a Collaborative Care Intervention for Patients with Hypertension (<https://doi.org/10.18865/ed.31.1.47>) | (Ibe et al., 2021) | Journal article | Identify how patients’ social determinants of health, such as health literacy and food insecurity, influence the engagement in a collaborative care intervention. | |
| Protocol for Responding to and Assessing Patients’ Assets, Risks, and Experiences (PRAPARE) Implementation and Action Toolkit  (<https://www.nachc.org/research-and-data/prapare/toolkit/>) | NACHC | Toolkit | Provide interested users with the resources,  best practices, and lessons learned to guide implementation, data collection, and responses to social  determinant needs. | |
| How to Collect Accurate and Complete Race / Ethnicity Data – A Step Toward Improving Health Equity  (<https://targetbp.org/tools_downloads/cme-course-how-to-collect-accurate-and-complete-race-ethnicity-data/>) | AHA/AMA Target: BP^TM^ | Webinar with free enduring CME/CE | Trains multi-disciplinary care team members on approaches to strengthen the collection of race and ethnicity demographic data. | |
| Race & Ethnicity Data Collection Essentials  (<https://targetbp.org/tools_downloads/race-ethnicity-data-collection-essentials/>) | AHA/AMA Target: BP^TM^ | Infographic about quality improvement and practice | Guides multidisciplinary care team members on approaches to strengthen the collection of race and ethnicity demographic data. | |
| August Webinar: Act Rapidly  (<https://www.heart.org/en/national-hypertension-control-initiative/community-health-center-hub>) | AHA | Webinar | Webinar geared towards Health Resources and Services  Administration-funded health centers to discuss the impact of treatment intensification on improving blood pressure control. | |

*Note.* References are on the last page of this document. ABC = Association of Black Cardiologists, Inc.; AHA = American Heart Association; AMA = American Medical Association; ASA = American Stroke Association; CDC = Centers for Disease Control and Prevention; MHI = Minority Health Institute, Inc.; NACHC = National Association of Community Health Centers; NINDS = National Institute of Neurological Disorders and Stroke; NMA = National Medical Association; PCNA = Preventive Cardiovascular Nurses Association

**Table S3.** Resource List Related to “Cultivate Community Supports”

| **Goal 2. Ensure that the places where people live, learn, work, and play support hypertension control.** | | | |
| --- | --- | --- | --- |
| ***Strategy A. Promote access to and availability of physical activity opportunities within communities.*** | | | |
| Resource (link) | Author/  Sponsor | Type of resource | Description |
| Move Your Way campaign (<https://health.gov/our-work/physical-activity/move-your-way-campaign>) | ODPHP | Campaign materials, community playbook, and partner promotion toolkits | Promotional campaign of the Physical Activity Guidelines for Americans (Guidelines: <https://health.gov/our-work/physical-activity/current-guidelines>). |
| Active People, Healthy Nation^SM^  (<https://www.cdc.gov/physicalactivity/activepeoplehealthynation/index.html>) | CDC | Initiative information, guidelines, education tools, and related links | A national initiative led by CDC to help people become more physically active by 2027. |
| Walk with a Doc (<https://walkwithadoc.org/>) | Walk with a Doc | Walking events newsletter, YouTube videos | Encourage people to increase their physical activities through participating in doctor-led walking groups. |
| GirlTrek (<https://www.girltrek.org/>) | GirlTrek | Walking events, blog, videos | A national health movement for African American women and girls through walking campaigns, community leadership, and health advocacy. |
| Exercise is Medicine® (<https://www.exerciseismedicine.org/>) | The American College of Sports Medicine | Action guide, provider materials, patient handouts | Make assessment and promotion of physical activity a standard in clinical care. |
| Physical Activity (<https://millionhearts.hhs.gov/tools-protocols/tools/physical-activity.html>) | Million Hearts® | Education tools, guidelines, related links | Encourage individuals and health care providers to increase physical activity and promote cardiovascular health. |
| CPSTF Findings for Physical Activity  (<https://www.thecommunityguide.org/content/task-force-findings-physical-activity>) | Community Preventive Services Task Force | Recommended interventions list | Review of the evidence for community interventions to increase physical activity. |
| ***Strategy B. Promote access to and availability of healthy food options within communities.*** | | | |
| Resource (link) | Author/  Sponsor | Type of resource | Description |
| Frederick Food Security Network (<https://www.hood.edu/academics/departments/department-biology/center-coastal-watershed-studies/frederick-food-security-network>) | Hood College | Newsletter, annual reports, YouTube video | A community gardening program improving food security for residents, reducing local water pollution, and promoting better eating habits. |
| Food Service Guidelines for Federal Facilities (<https://www.cdc.gov/obesity/downloads/guidelines_for_federal_concessions_and_vending_operations.pdf>) | CDC | Guideline | Provide standards for food, nutrition, environmental support, food safety, and behavioral design strategies in federal facilities. |
| VNAphilly - Partnering to end food insecurity  (<http://www.vnaphilly.org/2019/11/22/partnering-with-philabundance/>) | VNA Philadelphia | Partnership information | Partnerships helping address the significant food insecurity faced in communities. |
| Partnership for a Healthier America (<https://www.ahealthieramerica.org/>) | Partnership for a Healthier America | Event information, blog, videos, progress reports | Partnership leveraging the power of the private sector to make healthy food available in more areas in pursuit of health equity for people. |
| Life's Simple 7®  (Public: <https://www.heart.org/en/healthy-living/healthy-lifestyle/my-life-check--lifes-simple-7>  Workplace: <https://www.heart.org/en/professional/workplace-health/lifes-simple-7>) | AHA | Interactive online tool, including infographics, videos, and related links | Helps people assess and track their heart health information and gain a better understanding of their risk of heart disease and stroke. |
| ***Strategy C. Promote links between clinical services and community programs.*** | | | |
| Resource (link) | Author/  Sponsor | Type of resource | Description |
| Stanford’s Chronic Disease Self-Management (CDSMP) (<https://swap.stanford.edu/20170911010144/http://www.selfmanagementresource.com/programs/small-group/chronic-disease-self-management/>) | Self-Management Resource Center | Self-management program | Chronic care model designed to be delivered small group workshops to teach self-management to people with chronic health problems. |
| National Diabetes Prevention Program (<https://www.cdc.gov/diabetes/prevention/index.html>) | CDC | Lifestyle change program, education tools, checklist, and related links | Information about preventing diabetes, including links to certified programs. |
| Living Well MD Evidence-Based Programs (<https://mdlivingwell.org/programs/>) | Maryland Living Well Center of Excellence | Wellness program information, videos, booklets, and infographics | A variety of wellness programs teaching people how to self-manage chronic conditions, prevent falls, and address numerous health issues. |
| OSU Million Hearts® Fellowship Model (<https://wellness.osu.edu/faculty-and-staff/million-hearts>) | The Ohio State University/ Million Hearts® | Million Hearts® educational modules, Million Hearts® clinic information, events | Educate students and health care professionals on the Million Hearts® initiative to increase screenings and improve cardiovascular health. |
| Million Hearts® Community Clinical Linkages Toolkit (<https://www.heart.org/en/professional/million-hearts/resources-and-messaging/million-hearts-collaboration-community-clinical-linkages-toolkit>) | AHA | Toolkit (infographics), related links | Assist public health practitioners with developing, implementing, and sustaining strong community-clinical linkages. |
| Community Clinical Linkages for the Prevention and Control of Chronic Diseases: A Practitioner’s Guide (<https://stacks.cdc.gov/view/cdc/79520>) | CDC | Practitioner’s guide | Guide public health practitioners on key strategies implementing community-clinical linkages that focus on adults to improve population health. |
| Blood Pressure Self-Monitoring Program (<https://www.ymca.org/what-we-do/healthy-living/fitness/self-monitoring>) | YMCA of the USA | Program information, video | Offer personalized support as participants develop the habit of monitoring their blood pressure. |
| Community Health Workers (CHW) Inclusion Checklist (<https://www.cdc.gov/dhdsp/pubs/toolkits/chw-checklist.htm>) | CDC | Checklist | Provide a general framework for public health practitioners to lead or assist in including CHWs and integrating the CHW scope of practice in health care settings. |

*Note.* AHA = American Heart Association; CDC = Centers for Disease Control and Prevention; ODPHP = Office of Disease Prevention and Health Promotion

**Table S4.** Resource List Related to “Optimize Patient Care”

| **Goal 3. Optimize patient care for hypertension control.** | | | |
| --- | --- | --- | --- |
| ***Strategy A. Advance the use of standardized treatment approaches and guideline-recommended care.*** | | | |
| Resource (link) | Author/  Sponsor | Type of resource | Description |
| Improving Blood Pressure Control for African Americans Roadmap  (<https://www.nachc.org/wp-content/uploads/2020/11/Roadmap-BPAA-Interactive.pdf>) | NACHC | A roadmap for improving blood pressure control for African American patients | A useful tool listing potential interventions to improve blood pressure control in categories of core versus enhancement interventions. Useful for assessing current activities and prioritizing next interventions. |
| Target: BP^TM^ Quick Start Guides based upon the M.A.P. Framework:  - Quick start guide to measure accurately  (<https://targetbp.org/tools_downloads/quick-start-guide-to-measure-accurately/>)  - Quick start guide to act rapidly  (<https://targetbp.org/tools_downloads/quick-start-guide-to-act-rapidly/>)  - Quick start guide to partner with patients  (<https://targetbp.org/tools_downloads/quick-start-guide-to-partner-with-patients/>) | AHA/AMA Target: BP^TM^ | Toolkit (infographics) | Quality improvement tool suite with practice assessment tools, professional education with free enduring CME/CE, practice resources, and patient education tools. |
| Undiagnosed Hypertension (<https://millionhearts.hhs.gov/tools-protocols/undiagnosed-hypertension.html>) | Million Hearts® | Estimator tool, related materials, and links | Tool for health systems to estimate the number of patients who could have hypertension and assess potential amount of underdiagnosis (uses older definition of hypertension). |
| CardiOH  (<https://www.cardi-oh.org/>) | CardiOH Ohio Cardiovascular and Diabetes Health Collaborative | Monthly newsletter, podcasts, and Project ECHO® virtual training model | A statewide initiative of health care professionals  who share knowledge to improve Medicaid  patient outcomes and eliminate health disparities in cardiovascular diseases and diabetes. |
| Hypertension - 2017ACC/AHA/AAPA/ABC/ACPM/AGS/APhA/ASH/ASPC/NMA/PCNA Guideline for the Prevention, Detection, Evaluation, and Management of High Blood Pressure in Adults (<https://pubmed.ncbi.nlm.nih.gov/29146535/>) | (Whelton et al., 2018) | Journal article/Guideline | Clinical practice guideline covering a general approach, screening, follow-up, principles of drug therapy and special populations, and quality improvement in management of high blood pressure. |
| Hypertension Medication Treatment Protocol  (<https://www.ama-assn.org/system/files/2020-11/hypertension-medication-treatment-protocol.pdf>) | AMA/MAP BP^TM^ | Infographic | Treatment protocol for adults without specific conditions, including a generic medication summary. |
| Hypertension Control Change Package (<https://millionhearts.hhs.gov/files/HTN_Change_Package.pdf>) | Million Hearts® | Quality improvement change package | A quality improvement tool with  specific interventions to improve hypertension control in outpatient clinical settings. |
| The 7-step self-measured blood pressure (SMBP) quick guide  (<https://www.ama-assn.org/delivering-care/hypertension/7-step-self-measured-blood-pressure-smbp-quick-guide>) | AMA | Guideline, training videos, and education materials | An evidence-based resource to help physicians and care teams start using SMBP, including links to practical implementation tools. |
| Self-Measured Blood Pressure Monitoring (SMBP) Implementation Toolkit (<https://www.nachc.org/wp-content/uploads/2020/12/SMBP-Toolkit_FINAL.pdf>) | Million Hearts®/NACHC | Toolkit, including SMBP Protocol Design Checklist | Help organizations to implement (SMBP) successfully into their care processes and workflows. |
| Hypertension Guideline Toolkit  (<http://aha-clinical-review.ascendeventmedia.com/books/aha-high-blood-pressure-toolkit/>) | AHA | Toolkit (booklet) | Help health care providers integrate the 2017 Hypertension Clinical Practice Guideline into their practices. |
| Hypertension Guidelines Course  (<https://pcna.net/online-course/new-hypertension-guidelines/>) | PCNA | Education course for nurses | Continuing education for nurses on the current national hypertension guidelines and the clinical application. |
| Million Hearts® June 2020 SMBP Forum: SMBP for pregnant and post-partum women  (<https://www.youtube.com/watch?v=o1v7QTERlXQ>) | Million Hearts®/NACHC | YouTube video | Presentations for clinical and public health professionals from experts in maternal hypertension care introducing need for, obstacles to, and examples of using SMBP to support maternal health. |
| ***Strategy B. Promote the use of healthcare teams to manage hypertension.*** | | | |
| Resource (link) | Author/  Sponsor | Type of resource | Description |
| Innovations in pharmacy - An Advanced Registered Nurse Practitioner-Community Pharmacist Team-Based Approach to Managing Hypertension in a Rural Community Pharmacy (<https://doaj.org/article/b86afdaa01c04a55aa6555bc501108ed>) | (Parker, Kelchen, & Doucette, 2015) | Journal article | Effective team-based approach, an Advanced Registered Nurse Practitioner – community pharmacist team-based collaborative model for managing hypertension. |
| Advancing Team-Based Care Through  Collaborative Practice Agreements - A Resource and Implementation Guide  for Adding Pharmacists to the Care Team (<https://www.cdc.gov/dhdsp/pubs/docs/CPA-Team-Based-Care.pdf>) | CDC | Guideline | Describes a collaborative practice agreement (CPA), collaborative care, and provides a template CPA for a hypertension and cardiovascular disease service. |
| Wiley Clinical Cardiology - Development of an entirely remote, non-physician led hypertension management program (<https://onlinelibrary.wiley.com/doi/full/10.1002/clc.23141>) | (Fisher et al., 2019) | Journal article | A home-based blood pressure control program using Bluetooth-enabled blood pressure devices run by non-physicians providing efficient, effective, and rapid control for hypertension management. |
| BMC family practice - Healthcare professional-led interventions on lifestyle modifications for hypertensive patients - a systematic review and meta-analysis. (<https://bmcfampract.biomedcentral.com/articles/10.1186/s12875-021-01421-z>) | (Treciokiene et al., 2021) | Journal article | A systematic review and meta-analysis of interventions on lifestyle modifications of hypertensive patients performed by health care professionals. |
| ***Strategy C. Empower and equip patients to use self-measured blood pressure monitoring and medication adherence strategies.*** | | | |
| Resource (link) | Author/  Sponsor | Type of resource | Description |
| SMBP QUICK START GUIDE  (<https://targetbp.org/tools_downloads/smbp-quick-start-guide/>) | AHA/AMA Target: BP^TM^ | Toolkit (infographics) | Quality improvement tool suite with practice assessment tools, professional education with free enduring CME/CE, practice resources, and patient education tools. |
| Self-Measured Blood Pressure (SMBP) Monitoring (<https://millionhearts.hhs.gov/tools-protocols/smbp.html>)  Self-Measured Blood Pressure Monitoring - ACTION STEPS for Clinicians (<https://millionhearts.hhs.gov/files/MH_SMBP_Clinicians.pdf>) | Million Hearts® | Webpage, including toolkit, guideline, infographic, related links | A webpage with numerous links to support SMBP and Action Guide for clinicians interested in establishing an SMBP program. |
| Validatebp.org (<https://www.validatebp.org/>) | AMA | Device list | US Blood Pressure Validated Device Listing |
| SMBP training video (<https://www.ama-assn.org/sites/ama-assn.org/files/2019-07/SMBP-Training-English.mp4>) | AMA | Training video (mp4) | A video for patients on how to self-measure their BP. |
| Release the Pressure Self-Measured Blood Pressure (SMBP) Training Video (<https://www.youtube.com/watch?app=desktop&v=AIoXWcOVn6A>) | AMA | Training YouTube video | A video for African American women on how to measure their blood pressure. |
| How to Use Your Home Blood Pressure Monitor (<https://www.youtube.com/watch?app=desktop&v=0tGyRJxbYpQ>) | NACHC | Training YouTube video | A video for patients on how to self-measure their blood pressure. |
| SMBP patient infographic: “How to measure your blood pressure at home” (<https://www.ama-assn.org/system/files/2020-11/smbp-infographic.pdf>) | AMA/MAP BP^TM^ | Infographic | Visualization of an accurate blood pressure measurement for individuals. |
| Monitoring your blood pressure at home  (<https://www.heart.org/en/health-topics/high-blood-pressure/understanding-blood-pressure-readings/monitoring-your-blood-pressure-at-home>) | AHA | Webpage, including education materials (infographics, videos),  fact sheets, and e-news | Overall information helps people to measure blood pressure at home, including information on validated devices, a heart healthy diet, and for pregnant women. |
| The Cuff Project  (<https://www.preeclampsia.org/order-cuff-kits>) | PREECLAMPSIA^TM^ FOUNDATION | BP cuffs and Patient education materials, Cuff Kit^TM^ information | A resource for providers that commit to providing women at highest risk of developing preeclampsia and other hypertensive disorders of pregnancy with a validated automatic BP device and educational tools. |
| ***Strategy D. Recognize and reward clinicians and health systems that excel in hypertension control.*** | | | |
| Resource (link) | Author/  Sponsor | Type of resource | Description |
| Million Hearts® Hypertension Control Champions  (<https://millionhearts.hhs.gov/partners-progress/champions/index.html>)  Million Hearts® Hospitals & Health Systems Recognition Program (<https://millionhearts.hhs.gov/partners-progress/hospitals-health-systems/index.html>) | Million Hearts® | Webpage, including events, applications, awardees, success stories | Information on two recognition programs. Includes success stories and application information for getting recognition. |
| Community Health Quality Recognition (CHQR) Badges (<https://bphc.hrsa.gov/program-opportunities/quality/badges>) | HRSA | Webpage, including data platform, related links | Quality improvement awards badges recognize Health Center Program awardees and Look-Alikes (LALs) that have made notable quality improvement achievements. |
| Target: BP^TM^ Recognition Program  (<https://targetbp.org/recognition-program/>) | AHA/AMA Target: BP^TM^ | Program information and data platform | Annual award program for healthcare organizations that prioritize blood pressure control, demonstrate evidence-based blood pressure activities, and achieve control rates ≥ 70%. |

*Note.* References are on the last page of this document. AHA = American Heart Association; AMA = American Medical Association; CDC = Centers for Disease Control and Prevention; HRSA = Health Resources and Services Administration; NACHC = National Association of Community Health Centers; PCNA = Preventive Cardiovascular Nurses Association

**Table S5.** Resource List for Accurate Blood Pressure Measurement

| ***Accurate blood pressure measurement*** | | | |
| --- | --- | --- | --- |
| Resource (link) | Author/  Sponsor | Type of resource | Description |
| Target: BP^TM^ Quick Start Guide based upon the MAP Framework:  Quick Start Guide to Measure Accurately  (<https://targetbp.org/tools_downloads/quick-start-guide-to-measure-accurately/>) | AHA/AMA Target: BP^TM^ | Toolkit (infographic) | Quality improvement tool suite with practice assessment tools, professional education with free enduring CME/CE, practice resources, and patient education tools. |
| AMA’s resource on taking an accurate BP needs to go in resources. The one graphic you need for accurate blood pressure reading (<https://www.ama-assn.org/delivering-care/hypertension/one-graphic-you-need-accurate-blood-pressure-reading>) | AMA | Infographic | Infographic depicting the effects of poor technique on blood pressure results. |
| AANP’s Accurate Blood Pressure Measurement (<https://www.youtube.com/watch?app=desktop&v=20ycQyu7o4E>) | AANP | YouTube video | Education video for health care providers demonstrating method for taking accurate blood pressure. |
| Hypertension - Measurement of Blood Pressure in Humans: A Scientific Statement From the American Heart Association (<https://www.ahajournals.org/doi/10.1161/HYP.0000000000000087>) | (Muntner et al., 2019) | Journal Article/Guideline | Practice guide covering key components in accurately measuring blood pressure, including validation protocols for blood pressure monitoring devices, ambulatory blood pressure monitoring procedures, home blood pressure monitoring. |

*Note.* References are on the last page of this document. AANP = American Association of Nurse Practitioners; AHA = American Heart Association; AMA = American Medical Association

**References**

Allen, J. K., Dennison-Himmelfarb, C. R., Szanton, S. L., Bone, L., Hill, M. N., Levine, D. M., . . . Anderson, K. (2011). Community Outreach and Cardiovascular Health (COACH) trial: A randomized, controlled trial of nurse practitioner/community health worker cardiovascular disease risk reduction in urban community health centers*. Circulation. Cardiovascular Quality and Outcomes*, *4*(6), 595–602. doi:10.1161/circoutcomes.111.961573

Artinian, N. T., Flack, J. M., Nordstrom, C. K., Hockman, E. M., Washington, O. G., Jen, K. L., & Fathy, M. (2007). Effects of nurse-managed telemonitoring on blood pressure at 12-month follow-up among urban African Americans. *Nursing Research*, *56*(5), 312–322. doi:10.1097/01.NNR.0000289501.45284.6e

Band, R., Morton, K., Stuart, B., Raftery, J., Bradbury, K., Yao, G. L., . . . McManus, R. J. (2016). Home and Online Management and Evaluation of Blood Pressure (HOME BP) digital intervention for self-management of uncontrolled, essential hypertension: A protocol for the randomised controlled HOME BP trial. *BMJ Open*, *6*(11), e012684. doi:10.1136/bmjopen-2016-012684

Bosworth, H. B., Olsen, M. K., McCant, F., Harrelson, M., Gentry, P., Rose, C., . . . Oddone, E. Z. (2007). Hypertension Intervention Nurse Telemedicine Study (HINTS): Testing a multifactorial tailored behavioral/educational and a medication management intervention for blood pressure control. *American Heart Journal*, *153*(6), 918–924. doi:10.1016/j.ahj.2007.03.004

Fisher, N. D. L., Fera, L. E., Dunning, J. R., Desai, S., Matta, L., Liquori, V., . . . Scirica, B. M. (2019). Development of an entirely remote, non-physician led hypertension management program. *Clinical Cardiology*, *42*(2), 285–291. doi:10.1002/clc.23141

Ibe, C. A., Alvarez, C., Carson, K. A., Marsteller, J. A., Crews, D. C., Dietz, K. B., . . . Cooper, L. A. (2021). Social determinants of health as potential influencers of a collaborative care intervention for patients with hypertension. *Ethnicity & Disease*, *31*(1), 47–56. doi:10.18865/ed.31.1.47

Kerry, S. M., Markus, H. S., Khong, T. K., Cloud, G. C., Tulloch, J., Coster, D., . . . Oakeshott, P. (2013). Home blood pressure monitoring with nurse-led telephone support among patients with hypertension and a history of stroke: A community-based randomized controlled trial. *CMAJ*, *185*(1), 23–31. doi:10.1503/cmaj.120832

McManus, R. J., Little, P., Stuart, B., Morton, K., Raftery, J., Kelly, J., . . . Yardley, L. (2021). Home and Online Management and Evaluation of Blood Pressure (HOME BP) using a digital intervention in poorly controlled hypertension: Randomised controlled trial. *BMJ* (*Clinical Research Ed*.), 372, m4858. doi:10.1136/bmj.m4858

Muntner, P., Shimbo, D., Carey, R. M., Charleston, J. B., Gaillard, T., Misra, S., . . . Wright, J. T., Jr. (2019). Measurement of blood pressure in humans: A scientific statement from the American Heart Association. *Hypertension*, *73*(5), e35–e66. doi:10.1161/hyp.0000000000000087

Ogedegbe, G., Plange-Rhule, J., Gyamfi, J., Chaplin, W., Ntim, M., Apusiga, K., . . . Cooper, R. (2018). Health insurance coverage with or without a nurse-led task shifting strategy for hypertension control: A pragmatic cluster randomized trial in Ghana. *PLoS Medicine*, *15*(5), e1002561. doi:10.1371/journal.pmed.1002561

Parker, C. P., Kelchen, S. L., & Doucette, R. W. (2015). An advanced registered nurse practitioner-community pharmacist team-based approach to managing hypertension in a rural community pharmacy. *INNOVATIONS in Pharmacy*, *6*(2), 1–7. https://doi.org/10.24926/iip.v6i2.384

Sarfo, F. S., Treiber, F., Gebregziabher, M., Adamu, S., Nichols, M., Singh, A., . . . Ovbiagele, B. (2019). Phone-based intervention for blood pressure control among Ghanaian stroke survivors: A pilot randomized controlled trial. *International Journal of Stroke, 14*(6), 630–638. doi:10.1177/1747493018816423

Spies, L. A., Bader, S. G., Opollo, J. G., & Gray, J. (2018). Nurse-led interventions for hypertension: A scoping review with implications for evidence-based practice. *Worldviews on Evidence-Based Nursing, 15*(4), 247–256. doi:10.1111/wvn.12297

Treciokiene, I., Postma, M., Nguyen, T., Fens, T., Petkevicius, J., Kubilius, R., . . . Taxis, K. (2021). Healthcare professional-led interventions on lifestyle modifications for hypertensive patients – A systematic review and meta-analysis. *BMC Family Practice, 22*(1), 63. doi:10.1186/s12875-021-01421-z

Whelton, P. K., Carey, R. M., Aronow, W. S., Casey, D. E., Jr., Collins, K. J., Dennison Himmelfarb, C., . . . Wright, J. T., Jr. (2018). 2017 ACC/AHA/AAPA/ABC/ACPM/AGS/APhA/ASH/ASPC/NMA/PCNA guideline for the prevention, detection, evaluation, and management of high blood pressure in adults: A report of the American College of Cardiology/American Heart Association Task Force on Clinical Practice Guidelines. *Hypertension, 71*(6), e13–e115. doi:10.1161/hyp.0000000000000065

Wright, K. D., Klatt, M. D., Adams, I. R., Nguyen, C. M., Mion, L. C., Tan, A., . . . Scharre, D. W. (2021). Mindfulness in motion and Dietary Approaches to Stop Hypertension (DASH) in hypertensive African Americans. *Journal of the American Geriatrics Society, 69*(3), 773–778. doi:10.1111/jgs.16947
